# Principles of enhanced recovery in gastrointestinal surgery

**DOI:** 10.1007/s00423-022-02602-9

**Published:** 2022-07-21

**Authors:** Didier Roulin, Nicolas Demartines

**Affiliations:** Department of Visceral Surgery, Lausanne University Hospital CHUV and University of Lausanne UNIL, Lausanne, 1011 Switzerland

**Keywords:** Gastrointestinal surgery, Enhanced recovery

## Abstract

**Background:**

To reduce the impact of surgery-related stress, enhanced recovery after surgery (ERAS) pathways have been developed since over 15 years with subsequent improved postoperative outcome. This multimodal and evidence-based perioperative approach has spread to all fields of gastrointestinal surgery, from esophagus, stomach, duodenum and pancreas, liver, small intestine and colon, and rectum, as well as for other specialties like vascular and cardia surgeries or neurosurgery, among others.

**Purpose:**

The aim of this state-of-the-art article is to assess current state of evidence on perioperative management specifically in gastrointestinal surgery, with a focus on surgery-related aspects, outcome benefit, and future directions.

**Conclusion:**

The surgical team must promote continuous improvement of the patient’s ERAS compliance to ensure optimal perioperative care. Everyday clinical practice should be performed according to latest evidence-based medicine and challenging surgical dogma. Moreover, the surgeon must lead and support a multidisciplinary and collaborative teamwork tailored to patient’s need especially with anesthetists and nursing staff.

## Overview

Enhanced recovery is a standardized and multidisciplinary perioperative pathway providing guidance for perioperative management. Its goal is to attenuate the catabolic response induced by surgical stress and to use all necessary elements to support functional recovery [1]. The application of the principles of enhanced recovery is based on latest evidence-based medicine to accelerate and improve postoperative rehabilitation and the management starts prior to the operation already.

Following the development of “fast-track” protocols in the 1990s which primarily focused on length of stay reduction, in 2001 that the concept and term of “enhanced recovery after surgery” (ERAS) was introduced initially by a group of academic colorectal surgeons, to better highlight the main goal which was the improvement of postoperative recovery and not only its speed [2]. The main philosophy behind ERAS is to bring together the various healthcare practitioners encountered by the patient during his entire perioperative journey allowing a homogeneous and patient-centered care. This multidisciplinary management includes surgeons and anesthetists, nursing staff and physiotherapist, nutritionist among others, and the patient himself.

**Fig. 1:**
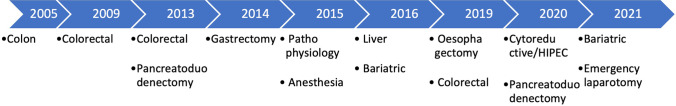
Enhanced recovery guidelines for gastrointestinal surgery

As illustrated on Fig. 1, after initial publication of the first ERAS guidelines for colorectal surgery in 2005 [3], several further recommendations for the pancreas [4], liver [5], bariatric [6], stomach [7], esophagus [8], cytoreductive [9, 10], and emergency surgeries [11] were published and updated [12, 13]. In addition, comprehensive guidelines on pathophysiology [14] and anesthesia [15] for gastrointestinal surgery were also established. Implementation of ERAS pathways was documented worldwide, with some national scaled diffusion for example in the Netherlands [16], UK [17], or Spain [18]. Most implementations were conducted from a bottom-up approach with a single unit starting its implementation. In addition, there was also some top-to-bottom approaches, with institutional-driven implementation like in the entire province of Alberta in Canada [19].

The present state-of-the art article will describe the actual status and benefits of ERAS in gastrointestinal surgery. As a detailed review of all elements for each type of surgery would be too broad and is already described in available textbook [20], this review will develop specific aspects related to the surgeon’s role and practice within ERAS.

## Multidisciplinary team and surgeon’s role

Implementing and sustainably running an ERAS pathway require a multidisciplinary team collaboration through all the patient’s journey. The surgeon plays an essential role in assuring that every healthcare professional involved is working not in silos, but in a collaborative longitudinal process. Teamwork and communication are essential to set common goals, and one of the most frequent barriers to ERAS implementation was reluctance to change from colleagues as suggested by a multinational survey [21]. Old habits, with sentences like “we always do so, why to change,” suggest how the comfort zone is important for surgeons and how change management has to be done with diplomacy but has to be based on data.

During formal training to implement an ERAS pathway in their respective hospital or unit [22], a multidisciplinary team is formed and composed by at least a dedicated nurse or physician assistant, an anesthetist, an administrator, and a surgeon. Other health care workers like physiotherapists, stomatherapists, or nutritionists are also of importance and may be invited to join the team. The team is then responsible to identify measurable goals, actions, and plans that are effectively put into practice. According to the Plan-Do-Study-Act [23] principles, regular assessment based on interactive audit system is conducted to allow standard reporting of clinical outcomes and quality improvement measures [24]. The sustainability of this multidisciplinary-driven improvement process was established in long-term ERAS follow-up studies [25]. The key is continuous audit of current practice, and in addition external validation of the dataset, including coverage, missing data, and accuracy are warranted [26].

A team leader, surgeon, or anesthetist needs to set and communicate clear goals and to hear and support all team members with the aim of achieving the best outcome in favor of the patients. Leadership is a complex and multifactorial issue and several types of leadership such as *authoritarian*, *adaptive*, *servant*, *situational*, *transactional*, and *transformational* can all be used in surgical setting [27]. On the contrary to aviation or nuclear energy industry, only few data are available in surgical field, but it has been shown that a more transformational (team-focused), as opposed to a transactional (task-focused), leadership in the operating room was associated with improved team behavior [28]. Thus, as a team leader, the surgeon must trust, motivate, and listen to all team members, as this will be key to increase team’s performance, and potentially patient’s outcome.

## Key elements of an enhanced recovery protocol

**Fig. 2:**
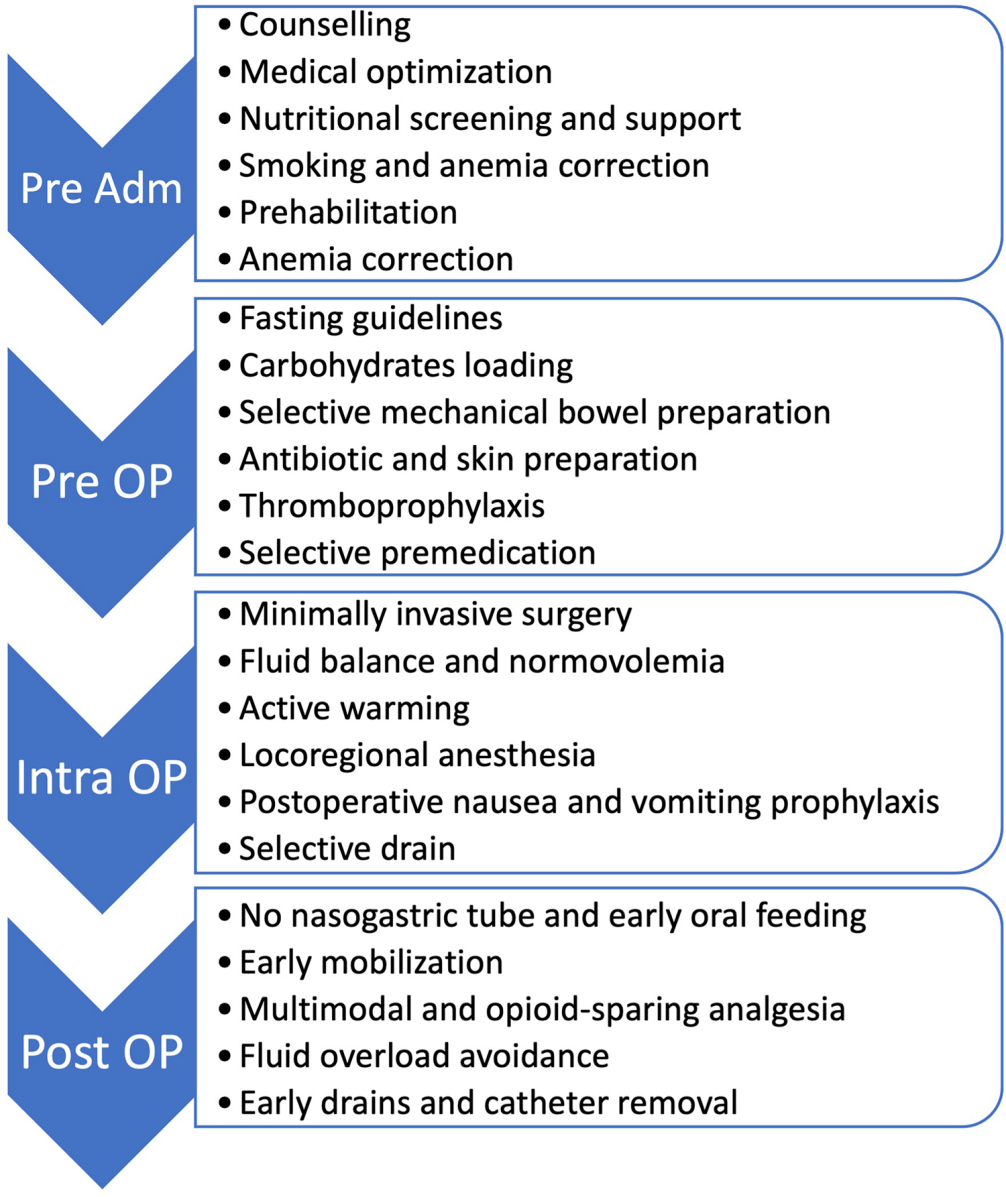
Summary of common enhanced recovery elements for gastrointestinal surgery. Adm: admission; OP: operative

An ERAS pathway usually gathers more than 20 specific elements, which should be applied during the pre-admission, pre-, intra-, and post-operative period. A summary of the most common elements for gastrointestinal surgery is provided on Fig. 2. Most of these ERAS elements are common across the different specialty, with some characteristic items base on the various subspecialties (hepato-pancreato-biliary, colorectal). However, all elements are aiming the same goal: minimize pathophysiological stress and improve the response to the surgical stress. The main targets are preoperative counseling and optimization, normovolemia, multimodal analgesia, and avoidance or early removal of tubes and drains, as well as early nutrition and early and active mobilization.

### Preoperative

In the preoperative phase, patient information and education are essential for their active participation in the early rehabilitation process. A detailed description of the surgical and anesthetic procedures, through personalized interviews, information brochures, and other multimedia means like website and videos (https://www.chuv.ch/fr/eras/eras-home/patients-et-famille/specialites/chirurgie-viscerale), reduces anxiety and promote postoperative recovery. In our institution, a 60-min preoperative specific information provided by the ERAS specialist nurse is performed in addition to the surgical visit. All patients are systematically screened for frailty and malnutrition, and malnourished patients with a nutritional risk score ≥ 3 are referred to specific dietician consultation. Smoking and alcohol cessation counseling is also offered. The administration of preoperative carbohydrate drinks, which reduces insulin resistance induced by surgical stress while reducing anxiety, is given the evening before the surgery (for example two sachet of Preload® 50 g) and up to 2 h (one sachet) before the procedure. In case of same day admission, the patient is instructed how to take those carbohydrate drinks at home.

### Intraoperative

Minimally invasive surgery and enhanced recovery after surgery have been two major developments in the last decades and are now both widely used in gastrointestinal surgery. The use of minimally invasive surgery is a technical issue requiring high-tech tools. Enhanced recovery after surgery, on the other hand, is more complex; it is a multimodal task requiring change management, with the need for reappraisal of the entire patient’s perioperative management. Whether the combination of both ERAS and minimally invasive surgery provides the best outcome remains surprisingly debated. As ERAS was initially developed for colorectal surgery, most data about the combination of ERAS and minimal invasive surgery (MIS) were obtained in this specialty. The first and robust proof of the added value of both ERAS and minimally invasive surgery was obtained from the LAFA-study group [29]. In this 9-center four-arm randomized-trial, patients undergoing colectomy were assigned to open vs laparoscopic surgery within either fast-track or standard care. The shortest postoperative stay was obtained in the laparoscopic enhanced recovery group, with similar postoperative morbidity. Further randomized trials, such as the multicenter collaborative EnRol study on colorectal cancer [30], or the randomized-study by Tiefenthal et al. [31] on right colectomy, also displayed shorter LOS when laparoscopy and ERAS were combined. As the principles of minimally invasive surgery have spread to most of gastrointestinal surgeries, further data are awaited in other sepcialties. For example, the ORANGE Segments trial, a multicenter international randomized controlled study, which will compare short- and long-term surgical and oncological outcomes between laparoscopic and open posterosuperior liver segment resections within an enhanced recovery program [32]. Several data strongly suggest that ERAS and MIS have synergistic effect in reducing complications and length of stay, and some new to be published ERAS guidelines, such as the updated ERAS liver guidelines expected to be published in 2022 and will include MIS as full part of ERAS programs (https://erassociety.org/specialty/hpb).

In addition, minimal and sparing use of drains is recommended. Prophylactic abdominal drainages are not recommended because their presumed value in terms of detection and prevention of intra-abdominal infections has not been proven. All nasogastric tubes are removed at the end of the operation, with the exception of eosophagectomy, because their use for postoperative prophylaxis increases the rate of atelectasis and pneumonia.

The anesthetic strategy will focus primarily on multimodal analgesia and fluid management. To avoid the side effects inherent to opiates such as nausea and paralytic ileus, postoperative analgesia should be opioid-sparing. The management of fluid homeostasis is, along with the prevention of metabolic stress, one of the key points of early rehabilitation. The reduction in fluid intake is significantly associated with fewer complications, particularly cardiopulmonary complications, and promotes tissue healing by reducing the risk of anastomotic insufficiency, parietal or cutaneous dehiscence, and wound infection. However, defining the right balance is challenging. To adapt as closely as possible to the homeostasis of each patient and to obtain the adequate range of normovolemia with cardiac output and tissue perfusion adequately maintained, several additional monitoring devices are available, for example, the pulse pressure variation and the stroke volume variation. The esophageal Doppler measures the variation in stroke volume.

### Postoperative

In the postoperative period, the objectives are a rapid recovery of intestinal function and a resumption of the patient’s autonomy. To guarantee volume management without excess intravenous fluids, patients are encouraged to drink starting 4 h after surgery. This allows to withdraw intravenous infusions the day after the operation at the latest. Early refeeding as soon as patient wakes up is encouraged and reduces the length of stay and complications. However, early refeeding can lead to more vomiting if it is not accompanied by systematic prevention of postoperative nausea and vomiting as well as multimodal management of paralytic ileus by stimulating early mobilization, reducing fluid intake intravenously, using oral laxatives (for example Magnesia San Pellegrino® twice a day), or even stimulating coffee consumption [33].

## Compliance

The individual impact of each ERAS elements on postoperative outcome is difficult to demonstrate and several studies were looking for which specific element would be determinant and if these more than 20 elements could be reduced. However, as initially described in the early stage of ERAS for colorectal surgery [34], the key of success is the overall number of applied elements, divided by the total number of ERAS elements, also called compliance. This was further confirmed in important multicentric studies for colorectal [35] and pancreatic surgery [36], where an increased compliance was related to a reduction in length of stay and a decrease of perioperative morbidity and costs.

## Outcome associated with enhanced recovery

### Clinical outcome

Several metrics are used as surrogate marker of the efficiency of an ERAS pathway. The first and historical metrics are length of stay (LOS) and readmission. However, these parameters are subjected to a high variability between different healthcare systems and may be influenced by a simple protocol effect. Then, additional metrics such as “ready-to-discharge” or “functional recovery” are also used. As ERAS mainly contributes to reduce postoperative stress, perioperative morbidity (expressed as percentage of complication or Comprehensive Complication Index [37]) is also a widely used metric.

For colorectal surgery, a recent meta-analysis displayed a reduction of 2.6 days for LOS, without increased readmission, and with a 34% decrease in perioperative morbidity [38]. However, the number of the ERAS elements used varied from 4 to 18 elements. Similar results were described for elective non colorectal major abdominal surgery [39] with a decrease in LOS of 2.5 days and in complication by 30% for patients treated within ERAS. There was also a significant reduction in time to first flatus of 0.8 days. Further meta-analysis revealed similar results for emergency laparotomy [40] and for pancreatoduodenectomy [41] with an associated reduction of delayed gastric emptying, as well as for cytoreductive surgery and hyperthermic intraperitoneal chemotherapy [42], liver resection [43], and even for liver transplantion [44] where a 55% reduction in intensive care unit was associated with the application of ERAS concept. A summary of all most recent meta-analysis for each surgical specialty is provided on Table 1. For all kind of surgery, a reduction in length of stay was observed without increased readmission (with the exception of gastrectomy where an increase of readmission was observed in the ERAS group). In most type of surgeries, a significant decrease of overall complications was observed, with the exception of upper gastrointestinal surgeries (bariatric [45], gastrectomy [46], oesophagectomy [47]) with, however, ERAS impact on minor or pulmonary complications. Most of the available evidence was graded as “low” and “very low” with mostly evaluated risks of bias, imprecision, and inconsistency. To allow rigorous and reproducible comparison and meta-analysis, each study reporting on ERAS should rely on the RECOvER (Reporting on ERAS Compliance, Outcome, and Elements Research) checklist [48] with a systematic report of the clinical pathway, the average compliance to each element, and the use of continuous audit.

**Table 1:** Summary of enhanced recovery meta-analysis for the different types of gastrointestinal surgeries with level of evidence

	Meta-analysis (1st author, year of publication)	Hospital length of stay (MD (95%*CI*))	LoE	Complications RR (95% *CI*)	LoE	Readmission RR (95% CI)	LoE
Colorectal	**Greer, 2018**	** − 2.6 (− 3.2, − 2.0)**	Low	**RR 0.66 (0.54, 0.80)**	Low	RR 1.1 (0.81–1.50)	Low
Pancreatoduodenectomy	**Kummerli, 2022**	** − 2.33 (− 2.98, –1.69)**	Moderate	**RD − 0.04 (− 0.08, − 0–01)**	Low	RR 0.02 (**− **0.01, 0.05)	Moderate
Gastrectomy	**Wee, 2019**	** − 2.47 (− 3.06, − 1.89)**	Low	RR 0.96 (0.75, 1.23)	Low	**RR 1.95 (1.03, 3.67)**	Low
Liver resection	**Noba, 2020**	** − 2.22 (− 2.77, − 1.68)**	Moderate	**RR 0.71 (0.65, 0.77)**	Low	RR 0.94 (0.70, 1.26)	Very low
Bariatric	**Zhou, 2021**	** − 1.11 (− 1.62, − 0.60)**	Low	OR 0.88 (0.75, 1.06)	Low	OR 0.84 (0.65, 1.08)	Low
Oesophagectomy	**Pisarska, 2017**	** − 3.55 (− 4.41, − 2.69)**	Low	RR 0.85 (0.71, 1.01)	Very low	RR 1.18 (0.89, 1.56)	Low
Cytoreductive	**Mao, 2021**	** − 2.82 (− 3.79, − 1.85)**	Low	RR 0.66 (0.41, 0.87)*	Very low	RR 0.55 (0.21, 1.49)	Very low
Emergency laparotomy	**Hajibandeh, 2020**	** − 3.09 (− 3.37, − 2.80)**	Low	**OR 0.50 (0.38, 0.66)**	Very low	RD − 0.01 (**− **0.04, 0.02)	Very low

Results in bold indicate statistically significant. *For Clavien grade III/IV. *MD*, median days; *LoE*, level of evidence according to GRADE; *RR*, risk ratio; *RD*, risk difference; *OR*, odds ratio

### Costs

When implementing an ERAS pathway, a significant investment in time and money is necessary to establish and audit the pathway, to train and build the dedicated team, and to allow continuous improvement of the process. But these costs are quickly overwhelmed by the return on investment in terms of improvement of postoperative outcome. First, the standardization of ERAS allows a reduction in unnecessary medication or laboratory testing [49]. Moreover, the reduction of complications has a major effect, as any complication is associated with a significant financial burden. This is in relation to the use of more medications, radiological investigation, and treatment, as well as prolonged length of stay. Finally, the reduction of length of stay allows freeing the bed earlier, which can be used to admit and treat other patients, a so-called cost of opportunity. The cost reduction was observed across all disciplines in gastrointestinal surgery with a constant return on investment [50]. Therefore, the implementation of ERAS represents the most favorable economic intervention, as the patient’s outcome is improved and the associated costs decreased. As the knowledge and implementation tools are available [51], it is the responsibility of each healthcare leader to convince administrators to support and even promote the application of ERAS principles.

### Long-term outcome

As detailed above, short-term beneficial outcome of ERAS is nowadays well-established. Surgical stress induces a local and systemic inflammatory response and impairs cellular immunity [52]; this may promote local and systemic spread of cancer cells. Any intervention reducing this perioperative stress could hamper the postoperative immunosuppression state, possibly leading to improved outcome. Consequently, the impact of ERAS on long-term outcome and especially oncological survival was assessed. The first initial evidence of this positive oncological impact was suggested in colorectal cancer patients in over 900 consecutive patients [53]. A compliance to ERAS protocol ≥ 70% was associated with a significant improved 5-year survival [53]. Nonetheless, two further studies focusing on rectal cancer [54] or pancreatic [55] cancer could not demonstrate any oncological survival benefit associated with increased compliance to ERAS. In a recent study based on a previous randomized trial of patients undergoing open liver resection with or without ERAS, the patient survival at 2 years was found to be significantly improved (91 vs 73%) with ERAS, and this advantage was even higher in cancer patients (91 vs 67%) [56]. On the other hand, however, this survival advantage was not reproduced at 5-year survival. A further appealing advantage of ERAS for oncological patients is that the improved postoperative recovery could allow a shorter time before initiation of postoperative adjuvant chemotherapy [57, 58]. And as such, faster start of chemotherapy might offer some oncological advantage but further data are awaited and this remains hypothetical for now.

## Future directions

### Mobile health and patient-reported outcomes

While the advantage of ERAS in gastrointestinal surgery is well-established for short-term outcomes, further data are still due for long-term and oncological outcome.

Patient’s experience and expectations from an ERAS pathway were poorly analyzed up to now but deserve further assessment. Careful evaluation of patient-reported outcomes (PROs), which focus on patient-reported symptoms, functional status, and quality of life (QoL), is advocated. To ease and follow patient’s perception, mobile health technology allowing PRO recording in real time will be paramount. First experiences with application-based mobile follow-up displayed high acceptance and economical benefits [59]. Mobile health follow-up allows clinicians to review longitudinal PRO reports and what could improve patients’ quality of life, enhance patient–clinician communication, reduce emergency department utilization, and lengthen survival [60]. To emphasize how PRO can be included within ERAS, the Perioperative Quality Initiative (POQI) workgroup detailed the incorporation of patient-centered PROs within enhanced recovery pathway [61]. Based on these recommendations, new data on PRO will provide more insight of what the benefits and expectations from a patient’s perspective are.

### Frailty

With the prolonged life expectancy, the number of elderly patients requiring major surgery is increasing. Perioperative management of elderly patients presents specific challenges related to their associated comorbidities and frailty [62]. Frailty is defined as reduced physiologic reserve to tolerate complications and has proven to predict unfavorable outcome, and there is an agreement to recommend a preoperative frailty screening [63]. Almost 60% of elderly patients were assessed as frail among surgical patients, and targeted care interventions such as delirium prevention and aspiration precautions allowed to reduce by almost 50% the 30-day postoperative complications [64]. Accordingly, it is of uttermost importance to develop frailty screening programs with multidisciplinary team involving geriatrician to ensure optimal care in elderly patient. However, which assessment tool has to be used and how many should be included in this evaluation are still under investigation and need to be further clarified, specifically within an ERAS pathway [65].

### Prehabilitation

Prehabilitation is a preoperative element aiming to increase the physiological reserve by optimizing cardiorespiratory capacity, muscle strength, and mental resiliency [66]. Thus, patients with low reserve and chronic medical conditions at high risk, such as elderly patients, are the most likely to benefit. A multimodal prehabilitation program encompasses tailored actions on physical, nutritional, and psychological aspects in the “window of opportunity” of the preoperative period. Prehabilitation is a promising and probably mandatory complement to ERAS, because common objectives, in addition to both ERAS and prehabilitation, require active involvement of the patient [67]. Notwithstanding, conflicting results from recent randomized studies failed to demonstrate clearly the added value of prehabilitation to ERAS, especially in colorectal surgery [68, 69]. In consequence, further data from multicentric large-scale trials on major gastrointestinal surgery are upcoming [70].

## Conclusion

The 15-year-old concept of enhanced recovery has undergone significant development with significant and convincing results since its deployment in gastrointestinal surgery. Enhanced recovery is a team effort that consists of implementing the principles based on evidence-based medicine in a standardized and systematic way. In this perioperative medicine, surgeons, anesthetists, and nursing staff have a key role that makes it possible to significantly improve the future of all surgical patients. Enhanced recovery should become gold standard in modern surgical perioperative management.
